# Identification of Timm13 protein translocase of the mitochondrial inner membrane as a potential mediator of liver fibrosis based on bioinformatics and experimental verification

**DOI:** 10.1186/s12967-023-04037-2

**Published:** 2023-03-10

**Authors:** Xiaomin Liao, Xianxian Ruan, Xianbin Wu, Zhejun Deng, Shanyu Qin, Haixing Jiang

**Affiliations:** 1 Department of Gastroenterology, The First Affiliated Hospital of Guangxi Medical University, 6 Shuangyong Road, Nanning, 530021 Guangxi China; 2 Department of Gastroenterology, The Third Affiliated Hospital of Guangxi Medical University, Nanning, 530000 Guangxi China

**Keywords:** Liver disease, Liver fibrosis, Translocase of inner mitochondrial membrane, Timm13, Hepatocytes

## Abstract

**Objective:**

To explore the association between translocase of the inner mitochondrial membrane 13 (Timm13) and liver fibrosis.

**Methods:**

Gene expression profiles of GSE167033 were collected from Gene Expression Omnibus (GEO). Differentially expressed genes (DEGs) between liver disease and normal samples were analyzed using GEO2R. Gene Ontology and Enrichment function were performed, a protein–protein interaction (PPI) network was constructed via the Search Tool for the Retrieval of Interacting Genes/Proteins (STRING), and the hub genes of the PPI network were calculated by MCODE plug-in in Cytoscape. We validated the transcriptional and post-transcriptional expression levels of the top correlated genes using fibrotic animal and cell models. A cell transfection experiment was conducted to silence Timm13 and detect the expression of fibrosis genes and apoptosis genes.

**Results:**

21,722 genes were analyzed and 178 DEGs were identified by GEO2R analysis. The top 200 DEGs were selected and analyzed in STRING for PPI network analysis. Timm13 was one of the hub genes via the PPI network. We found that the mRNA levels of Timm13 in fibrotic liver tissue decreased (P < 0.05), and the mRNA and protein levels of Timm13 also decreased when hepatocytes were stimulated with transforming growth factor-β1. Silencing Timm13 significantly reduced the expression of profibrogenic genes and apoptosis related genes.

**Conclusions:**

The results showed that Timm13 is closely related to liver fibrosis and silencing Timm13 significantly reduced the expression of profibrogenic genes and apoptosis related genes, which will provide novel ideas and targets for the clinical diagnosis and treatment of liver fibrosis.

**Supplementary Information:**

The online version contains supplementary material available at 10.1186/s12967-023-04037-2.

## Background

Liver fibrosis is a common pathological feature of chronic liver disease, and is characterized by the gradual replacement of functional liver tissue by highly cross-linked extracellular matrix rich in type I/III collagen. Fibrosis is considered to be a precancerous state, which provides an appropriate microenvironment for tumor development. The main causes of liver fibrosis include chronic viral infection, alcoholism, fatty liver, biliary diseases, autoimmune diseases, metabolic etiology, iron or copper overload, nonalcoholic steatohepatitis and toxicant exposure [1]. Hepatocytes (HCs) are the most common liver cells, accounting for about 80% of total liver cells, and have important functions. During the occurrence and development of liver fibrosis, hepatocyte apoptosis increases significantly. Although HCs are not the main cell source of extracellular matrix in liver tissue, HCs can activate hepatic stellate cells (HSCs) in the event of inflammation and necrosis, which can be used as the initiating factor of liver fibrosis and promote the occurrence and development of liver fibrosis. A large number of experimental results show that HCs produce malondialdehyde after continuous stimulation, causing the activation of HSCs [2–4]. The liver has abundant mitochondria [5], and in the process of understanding the pathogenesis of liver diseases, more and more researchers have studied mitochondrial function. Horn et al. found that hepatocyte free cholesterol overload can lead to endoplasmic reticulum stress, mitochondrial dysfunction, production of toxic oxysterols and cholesterol crystallization in lipid droplets, which can lead to hepatocyte apoptosis, necrosis or pyroptosis, and activation of HSCs leading to liver fibrosis [6]. Li et al. found that the use of carbon tetrachloride (CCl4) or acetaminophen in cultured mouse primary hepatocytes can lead to mitochondrial dysfunction, the release of mitochondrial DNA from damaged hepatocytes to adjacent hepatocytes and HSCs through extracellular vesicles, and mediate activated hepatocyte injury and fibrosis, and pretreatment of mouse primary hepatocytes with tetramethylpyrazine can prevent these pathological effects[7]. Nwaechefu et al. found in a rat model induced by CCl4 that *Cajanus cajan* can protect against liver injury by inhibiting the opening of mitochondrial permeability transition pores, preventing CCl4-induced liver oxidative stress and inhibiting the inflammatory reaction [8]. The study of a nonalcoholic steatohepatitis mouse model indicated that nobiletin reduced hepatocyte death, liver inflammation and fibrosis by regulating liver oxidative stress and mitochondrial dysfunction [9]. To date, increasing studies have provided significant evidence to confirm that mitochondrial dysfunction plays an important role in liver diseases and mitochondrial targeting therapy may be a promising treatment for liver diseases [10, 11]. Most mitochondrial proteins are encoded in the nucleus, in the mitochondrial inner membrane, the inner membrane transposase (Timm) 23 complex facilitates import into the matrix, and the Timm8p-Timm13p complex promotes the translocation of transmembrane space by binding to the membrane spanning domains [12]. A previous study suggested that Timm13 is highly expressed in the brain and liver [13]; however, there are no reports on the role of Timm13 in liver fibrosis nationally and internationally, and there are few reports on the role of Timm13 in other diseases. In the present study, we used bioinformatics to analyze significant liver fibrosis-related genes, and then explored the relationship between Timm13 and liver fibrosis using cellular experiments and animal models. Finally, we examined the mechanism of Timm13 in regulating liver fibrosis by targeted knockdown of Timm13 (Additional file 1: Fig. S1). Our aim was to identify a viable target for the diagnosis and treatment of liver fibrosis.

## Methods

### Data collection

The gene expression profiles of the GSE167033 dataset which included 46 liver tissues of mice that had been treated with CCl4 at different time points (2 and 8 h, 1, 2, 4, 6, 8, and 16 days following CCL4 administration) were collected from the Gene Expression Omnibus (GEO) database (https://www.ncbi.nlm.nih.gov/geo/). The details of the GSE167033 dataset are listed in Additional file 2: Table S1.

The samples were divided into the case group (with 16 days CCl4 treatment) and the control group (without CCl4 treatment). Our previous study found that 2 weeks after CCl4 was intraperitoneally injected into mice, fibrosis related changes occurred in hepatocytes, liver tissues and gene expression [14]. Therefore, we chose the sample with the longest 16 days of administration in the dataset as the liver fibrosis case group.

### Analysis of differentially expressed genes (DEGs) in liver fibrosis

Gene differences analysis was performed (case vs control) in GEO2R (https://www.ncbi.nlm.nih.gov/geo/geo2r/?acc), and the liver fibrosis standards were set as log fold change |logFC|> 1.5 and P < 0.05. There are 44,923 probe numbers listed in the GPL1261 platform, after mapping the probe into the gene, there were 21,722 genes in the results. GEO2R was used to plot the volcano of DEGs and the diagram of the intersection of DEGs and liver fibrosis. The DEGs were used for subsequent analysis [15].

### GO functional enrichment and KEGG analysis of liver fibrosis-related differential genes

The potential functional enrichment of liver fibrosis-related differential genes was explored using the MCODE plug-in in Cytoscape. A P < 0.05 was considered a significant enrichment function.

### Construction of the protein–protein interaction (PPI) network

According to the GEO2R sequencing results, the top 200 DEGs with the highest correlation were selected from 21,722 genes and were used to construct a PPI network using the Search Tool for the Retrieval of Interacting Genes/Proteins (STRING) database, and the hub genes (top 4) of the PPI network were calculated using the ClueGO and MCODE plug-in in Cytoscape software (version 3.8.2; https://cytoscape.org) [15].

### CCl4-induced liver fibrosis model

Normal male BALB/c mice, aged 6 weeks and initially weighing 18 − 20 g, were purchased from the Laboratory Animal Center (Guangxi Medical University, Nanning, China). All animals received humane care. All experimental procedures on mice were approved by the ethics committee of The First Affiliated Hospital of Guangxi Medical University. The animals were housed in a controlled environment (12 h light/dark cycle; temperature: 22 − 24 °C) and received water ad libitum in the Animal Care Facility Service (Guangxi Medical University). The mice were divided into three groups, with six mice in each group: (1) the mice in group 1 were control animals and received a vehicle (olive oil); (2) the mice in group 2 were injected intraperitoneally with CCl4 (Sigma-Aldrich, St. Louis, MO, USA) (0.1 mL of a solution containing 20 g of CCl4 dissolved in olive oil at a 1:10 ratio) three times per week for 4 or 6 weeks to induce liver fibrosis; and (3) the mice in group 3 were injected intraperitoneally with CCl4 (0.1 mL of a solution containing 20 g of CCl4 dissolved in olive oil at a 1:10 ratio) three times per week for 4 or 6 weeks to induce liver fibrosis. All mice were killed under light ether anesthesia 72 h after the final dose of CCl4 or olive oil. The liver was immediately removed. All samples were kept on ice until analysis. First, the liver was cut into fragments. Then, liver samples were either stored in formaldehyde or snap-frozen in liquid nitrogen and stored at − 80 °C.

### Histological and immunochemical analyses

Tissue sections were prepared at a thickness of 4 μm and stained with hematoxylin and eosin according to standard procedures. Two experienced pathologists, who were blinded to the experimental details, assessed liver histology using an Eclipse E800 Microscope (Nikon, Kawasaki, Japan). Ishak scores (a liver fibrosis scoring system) were then determined for each tissue section.

### Cell culture

AML12 mouse hepatocytes, which are immortalized normal mouse hepatocytes, were purchased from WHELAB (Shanghai, China). The cells were cultured in Dulbecco’s Modified Eagle’s Medium (Gibco, Gaithersburg, MD, USA) supplemented with 1% ITS Liquid Media Supplement, 40 ng/mL dexamethasone, 10% heat-inactivated fetal bovine serum (BI, Israel; VivaCell, Shanghai, China), 1% penicillin/streptomycin, and bicarbonate at 37 °C under 5% CO_2_. AML12 cells were divided into five groups: control, transforming growth factor beta1 (TGF-β1, Novoprotein, Suzhou, China) at concentrations of 5, 10, and 20 ng/mL. Each treatment was for an additional 48 h.

### CCK-8 assay

Proliferation was monitored by CCK-8 assays (Meilun Biotechnology, Dalian, China). In brief, cells were inoculated into 96-well plates and cultured for 24 h at 37 °C. Then, 10 µL of CCK-8 enhanced solution was added to each well and incubated for 1.5 h at 37 °C. The absorbance at 450 nm was then determined with a microplate reader and each group was allocated three wells. All experiments were performed in triplicate.

### Cell transfection

Silencing of Timm13 in AML12 cells was achieved by the transfection of cells with a SiRNA (Sangon Biotech, Shanghai, China) with the Advanced DNA RNA Transfection Reagent™ (Zeta Life, Menlo Park, CA, USA) in accordance with the manufacturer’s protocol. In brief, AML12 cells were placed on the surface of culture plates 1 day in advance and allowed to grow to 60 − 80% confluency. Then, the plasmid was directly mixed with transfection reagent (1:1) and mixed by pipette (10–15 times). Following incubation at room temperature for 15 min, the complex was added to the cell culture plates, mixed gently, and incubated in a CO_2_ incubator for 48 h.

### RNA extraction and real-time polymerase chain reaction (RT-PCR)

Total RNA was isolated from Raw 246.7 cells by homogenizing liver tissues using a NucleoZOL isolation kit (740404.6, Macherey–Nagel, Düren, Germany) in accordance with the manufacturer’s protocol. RT-PCR assays were performed using Prime ScriptTM RT Master Mix (Perfect Real Time) reagent kits (RR036A, TaKaRa Bio, Shiga, Japan), along with a FastStart Universal SYBR Green Master (ROX) kit (4913914001, Roche, Mannheim, Germany), according to the manufacturer’s instructions. The primers used were as follows: mouse GAPDH, (forward) 5′-GGT TGT CTC CTG CGA CTT CA-3′ and (reverse) 5′-TGG TCC AGG GTT TCT TTA CTC C-3′; mouse Timm13, (forward) 5′-GAA GAG AGT GAG GAC CCG ACA GAG-3′ and (reverse) 5′-GAG GTG ACA CGC CTG CTT TAC TG-3′; mouse α-SMA (alpha smooth muscle actin), (forward) 5′-CGT GGC TAT TCC TTC GTG ACT G-3′ and (reverse) 5′-CGT CAG GCA GTT CGT AGC TCT TC-3′; mouse COL-1 (collagen I), (forward) 5′-GAC AGG CGA ACA AGG TGA CAG AG-3′ and (reverse) 5′-CAG GAG AAC CAG GAG AAC CAG GAG-3′; mouse MMP9 (matrix metalloprotein 9), (forward) 5′-CAA AGA CCT GAA AAC CTC CAA C-3′ and (reverse) 5′-GAC TGC TTC TCT CCC ATC ATC-3′; mouse TIMP1 (TIMP metallopeptidase inhibitor 1), (forward) 5′-GCA AAG AGC TTT CTC AAA GAC C-3′ and (reverse) 5′-CTC CAG TTT GCA AGG GAT AGA T-3′; mouse BAX (BCL2-Associated X), (forward) 5′-TTG CCC TCT TCT ACT TTG CTA G-3′ and (reverse) 5′-CCA TGA TGG TTC TGA TCA GCT C-3′; mouse BAD (Bcl-2-associated death protein), (forward) 5′-GAA GAC GCT AGT GCT ACA GAT A-3′ and (reverse) 5′-CTG CTG ATG AAT GTT GCTC C-3′. The PCR conditions were as follows: one cycle of 50 °C for 2 min, and 95 °C for 10 min, 40 cycles of 15 s at 95 °C, and 1 min at 60 °C. The expression of the target gene mRNA was normalized to that of GAPDH. All reactions were performed in triplicate for each sample. At least three independent experiments were carried out for each experimental condition.

### Western blotting

RIPA buffer (Solarbio, Shanghai, China) was used to lyse cells and a BCA kit (Beyotime, China) was used to quantify protein levels. β-Actin (AbMART, Shanghai, China) was used as a loading control. The primary antibody specific for Timm13 was obtained from NOVUS (USA; NBP2-13431). Anti-rabbit and anti-mouse secondary antibodies were obtained from Invitrogen (Carlsbad, USA). An Odyssey two-color infrared laser imaging system (LI-COR Biosciences, Lincoln, NE, USA) was employed to scan the blots. The quantitative analysis of grey values was performed using ImageJ software (NIH, USA).

### Statistical analysis

Data are presented as the mean ± Standard Deviation of triplicate independent experiments. SPSS, version 25.0 (SPSS, Chicago, IL, USA) and GraphPad Prism 9 (GraphPad software) was utilized for all statistical analysis. Data were compared with the Student’s *t*-test and one-way analysis of variance (ANOVA). Probability (P) < 0.05 were considered statistically significant.

## Results

### Screening of DEGs

**Fig. 1:**
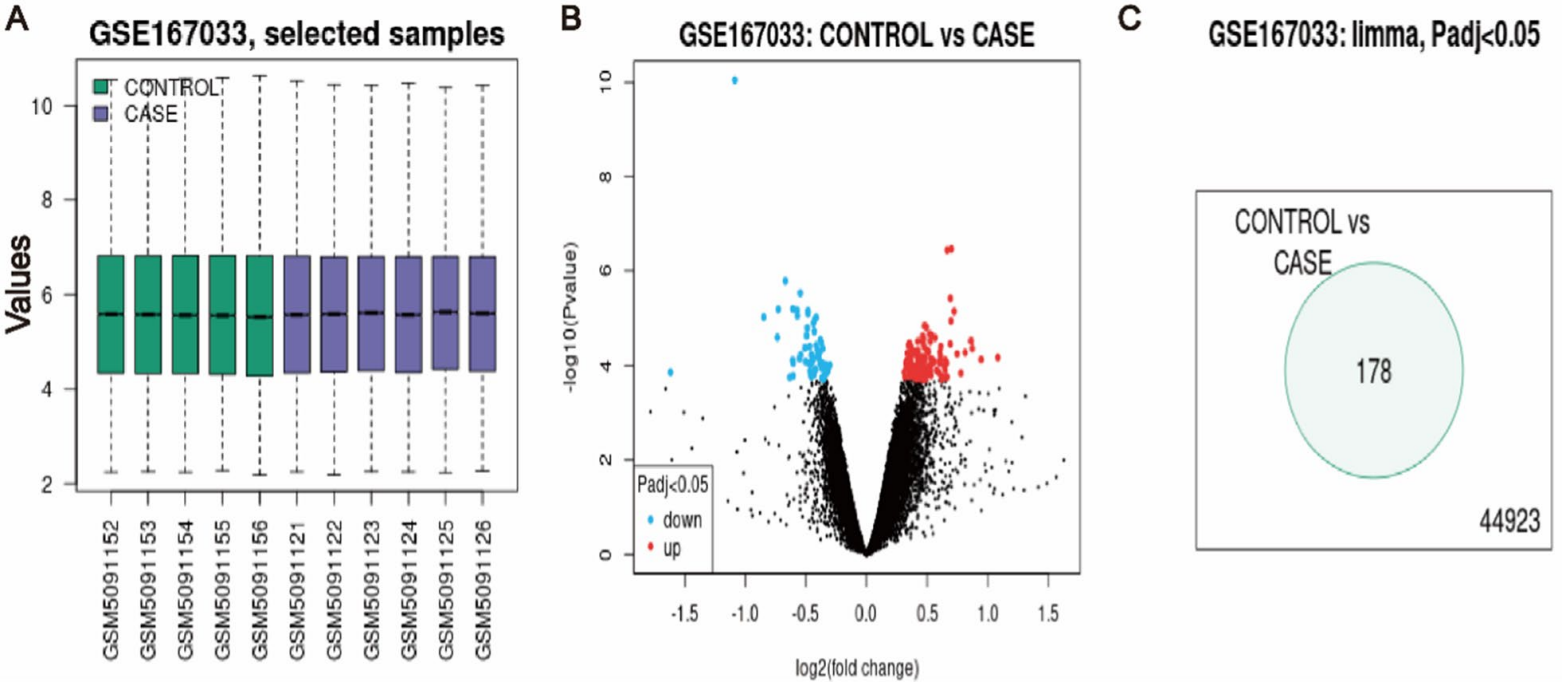
Screening of DEGs. **A** Boxplot of the GSE167033 dataset. **B** Volcano plot of the GSE167033 dataset. **C** Venn diagram of the DEGs. *DEGs* differentially expressed genes

The GSE167033 datasets were used to screen the DEGs between normal control and liver fibrosis samples (16 days CCl4 administration). The datasets contained two groups of data, including six groups of samples in the case group (16 days CCl4 administration) and five groups of samples in the control group (Fig. 1A, B), 178 DEGs were identified based on the selected criteria (Fig. 1C).

### GO functional enrichment and KEGG analysis of liver fibrosis-related DEGs

Results of the liver fibrosis-related DEGs function analysis showed that the biological processes of these genes mainly related to mitochondria, nucleobase metabolic process, vascular endothelial growth factor receptor signaling pathway, cardiac conduction, regulation of glucose transmembrane transport, DNA replication, cytoplasmic microtubule organization, regulation of microtubule polymerization, and regulation of microtubule depolymerization (Table 1). In addition, KEGG analysis did not enrich any pathway.

**Table 1:** The GO analysis of liver disease-related DEGs

	A	B	C	D	E	F
1	ID	Term	Ontology Source	Tem P value	Tem P value	Group P value
2	GO:0006626	Protein targeting to mitochondrion	Biological Process	0.00	0.02	0.00
3	GO:0009112	Nucleobase metabolic process	Biological Process	0.00	0.02	0.00
4	GO:0048010	Vascular endothelial growth factor receptor signaling pathway	Biological Process	0.01	0.02	0.01
5	GO:0061337	Cardiac conduction	Biological Process	0.01	0.01	0.01
6	GO:0,010,828	Positive regulation of glucose transmembrane transport	Biological Process	0.01	0.02	0.01
7	GO:0044786	Cell cycle DNA replication	Biological Process	0.00	0.02	0.01
8	GO:0033260	Nuclear DNA replication	Biological Process	0.00	0.02	0.01
9	GO:0090329	Regulation of DNA-dependent DNA replication	Biological Process	0.01	0.02	0.01
10	GO:0031122	Cytoplasmic microtubule organization	Biological Process	0.01	0.01	0.03
11	GO:0031110	Regulation of microtubule polymerization or depolymerization	Biological Process	0.01	0.02	0.03
12	GO:0046785	Microtubule polymerization	Biological Process	0.01	0.02	0.03
13	GO:0031112	Positive regulation of microtubule polymerization or depolymerization	Biological Process	0.00	0.02	0.03
14	GO:0031113	Regulation of microtubule polymerization	Biological Process	0.00	0.01	0.03
15	GO:0031116	Positive regulation of microtubule polymerization	Biological Process	0.00	0.02	0.03

### Construction of the PPI network

**Fig. 2:**
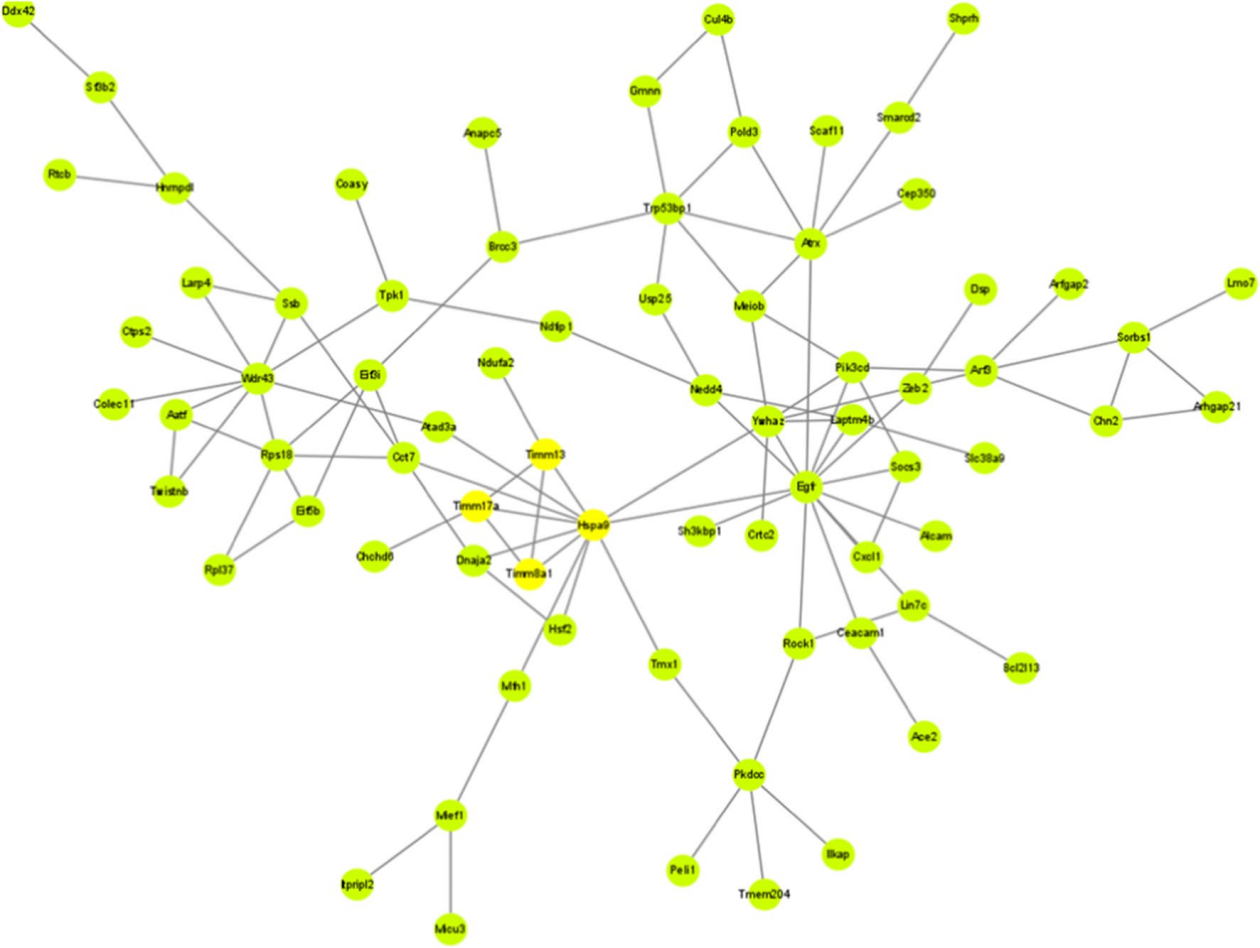
PPI network of DEGs. The protein–protein interaction network of the DEGs. *PPI* protein–protein interaction, *DEGs* differentially expressed genes

**Fig. 3:**
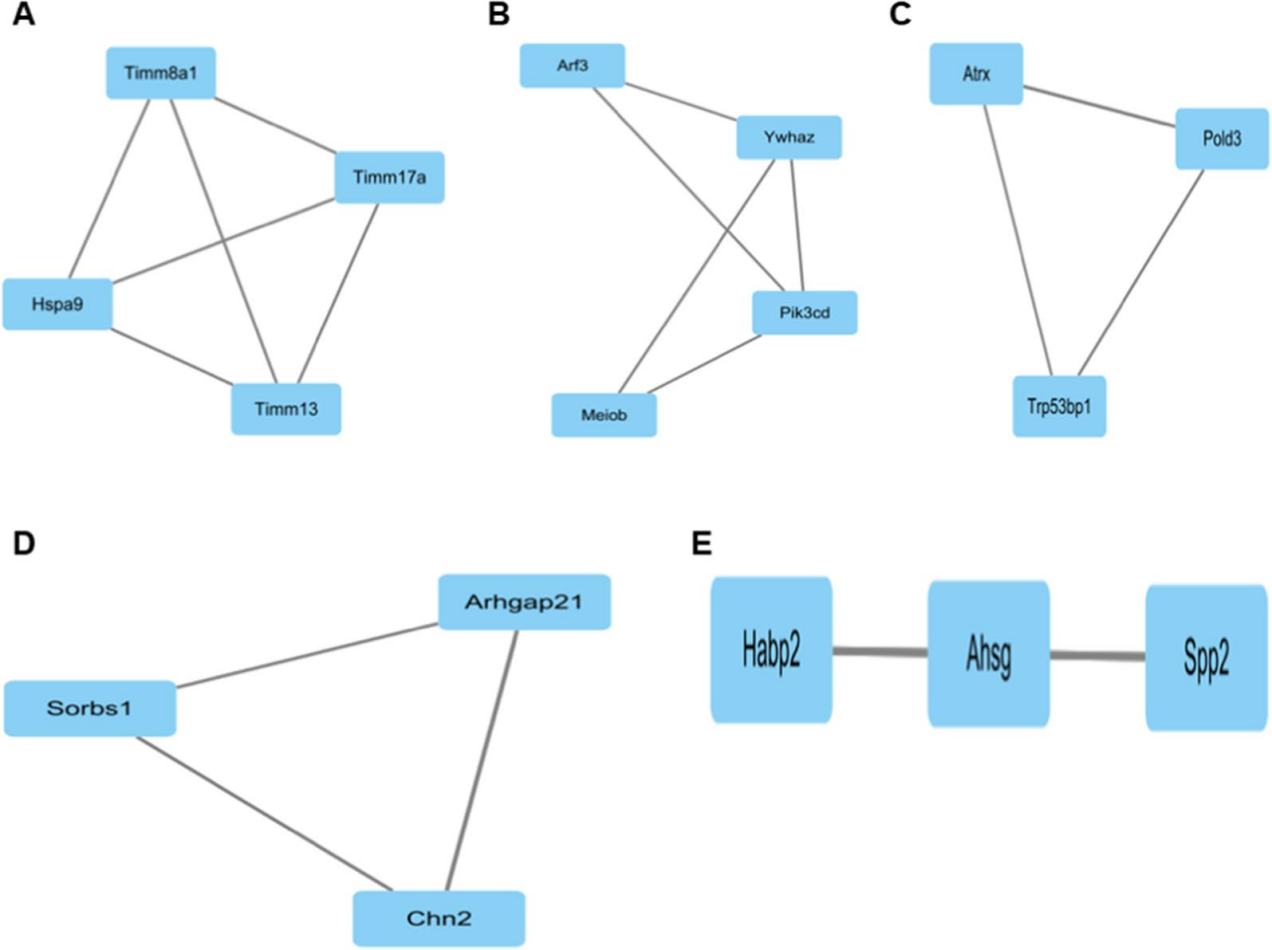
Cluster network of the DEGs. DEGs, differentially expressed genes

To explore the relationship of DEGs, we used the STRING database to construct a PPI network. According to the GEO2R sequencing results, the top 200 genes with the highest correlation were selected from 21,722 genes and analyzed in STRING, and the hub genes of the PPI network were calculated by the MCODE plug-in in Cytoscape (Fig. 2). Through the MCODE plug-in of Cytoscape, five cluster networks were obtained. The scores from high to low were cluster network A (including Timm8a1, Timm17a, Timm13 and Hspa9), cluster network B (including Arf3, Ywhaz, Pik3cd, and Meiob), cluster network C (including Atrx, Pold3, and Trp53bp1), cluster network D (including Sorbs1, Arhgap21, and Chn2), and Cluster network E (including Habp2, Ahsg, and Spp2) (Fig. 3 A–E). As the role of Timm13 in liver fibrosis was not reported, we therefore selected Timm13 for the following experiments.

### Liver fibrosis in vivo and in vitro validation

**Fig. 4:**
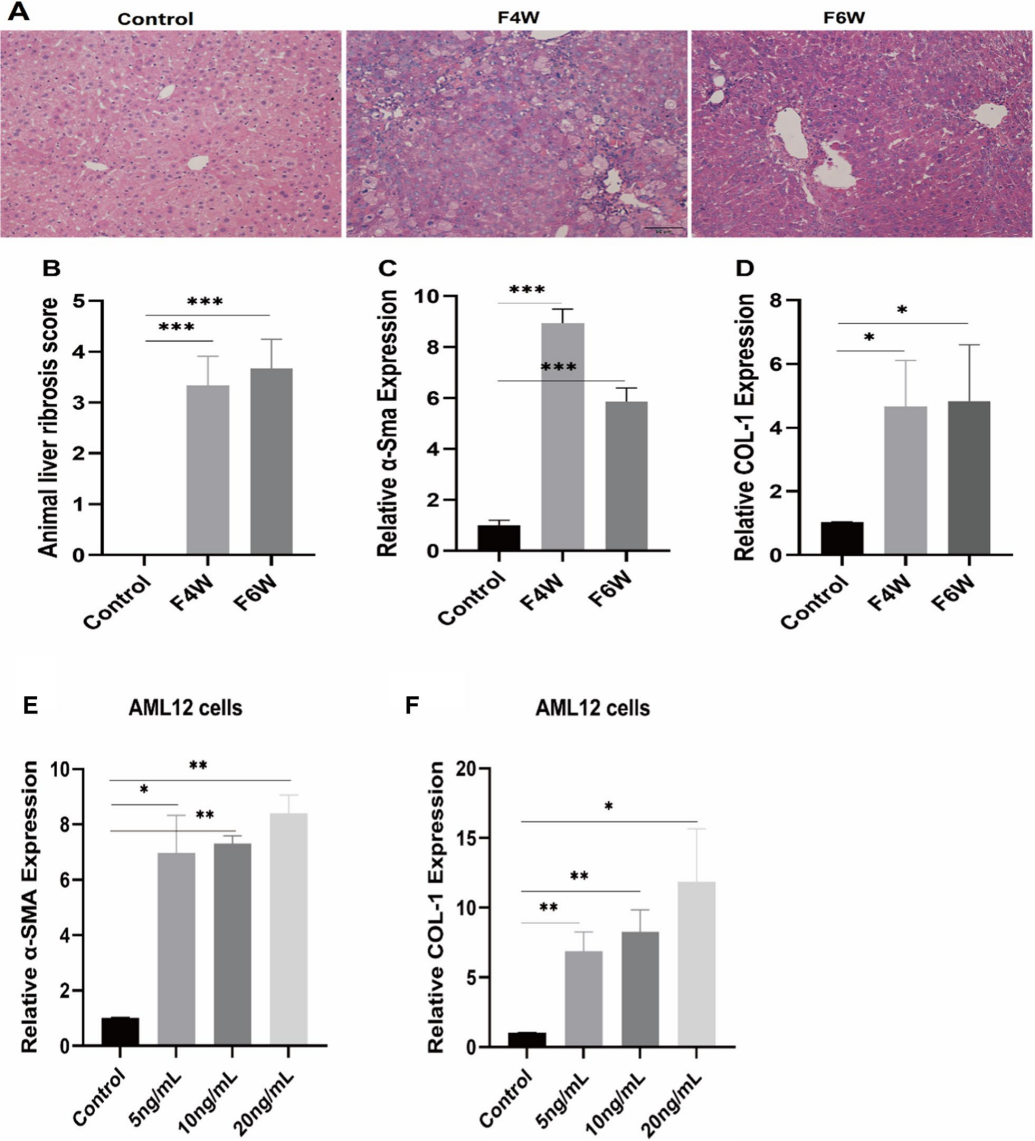
Evaluation of fibrosis in liver tissues. **A** Histological images of mouse livers stained with hematoxylin and eosin (50 μm). **B** Ishak liver fibrosis scores. **C** The relative expression levels of α-SMA in liver tissues. **D** The relative expression levels of COL-1 in liver tissues. **E** The relative expression levels of α-SMA in AML12 hepatocytes. **F** The relative expression levels of COL-1 in AML12 hepatocytes

Histological images of mouse livers stained with hematoxylin and eosin showed that CCl4 induced liver fibrosis which was obvious at 4W and 6W. The relative expression levels of α-smooth muscle actin (α-SMA, P < 0.001) and collagen 1 (COL-1, P < 0.05) in liver tissues from mice treated with CCl4 at 4 and 6 weeks were significantly increased (Fig. 4A–D; *P < 0.05, **P < 0.01, ***P < 0.001). In order to construct a cellular fibrosis model, AML12 mouse hepatocytes were stimulated with different concentrations of TGF-β1, and the fibrosis-related genes of α-SMA and COL-1 were verified at the mRNA level. The relative expression levels of α-SMA and COL-1 in AML12 cells were significantly increased (Fig. 4E and F; *P < 0.05, **P < 0.01).

### Cell proliferation

**Fig. 5:**
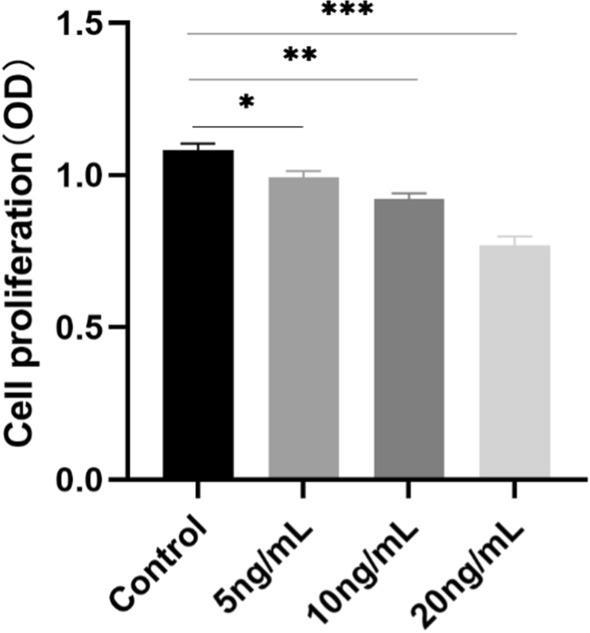
The effect of TGF-β1 on cell proliferation. The effects of different concentrations of TGF-β1 on AML12 cell proliferation

We tested the effects of TGF-β1 on AML12 cell proliferation using the CCK8 assay and found that TGF-β1 inhibited the proliferation of AML12 cells in a concentration-dependent manner. When TGF-β1 increased, cell proliferation decreased (Fig. 5; *P < 0.05, **P < 0.01, ***P < 0.001).

### Validation of Timm13 messenger RNA expression in vivo and in vitro

**Fig. 6:**
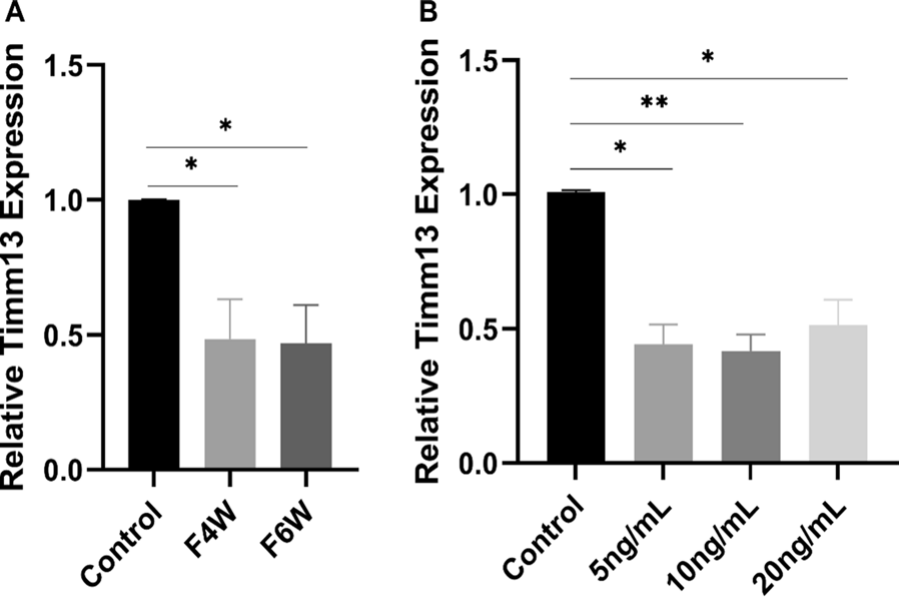
Validation of mRNA transcriptional levels via rat models and cell models. **A** Expression levels of Timm13 in different stages of fibrotic liver tissues. **B** Timm13 expression level in AML12 hepatocytes stimulated with different concentrations of TGF-β1. mRNA, messenger RNA

We detected Timm13 in fibrotic liver tissues and hepatocytes at the mRNA level, and our results indicated significant downregulation of Timm13 mRNA in liver fibrosis models and cell models (F4W, P = 0.0414; F6W, P = 0.0365; 5 ng/mL, P = 0.0119; 10 ng/mL, P = 0.0078; 20 ng/mL, P = 0.024) (Fig. 6; *P < 0.05, **P < 0.01). From the results of fibrotic liver tissue, the expression level of Timm13 decreased gradually with the progression of fibrosis, but there was no significant correlation between the expression level of Timm13 and the concentration of TGF-β1 in the cell experiment. This may be attributed to the differences between in vivo and in vitro experiments.

### Validation of Timm13 protein expression in vitro

**Fig. 7:**
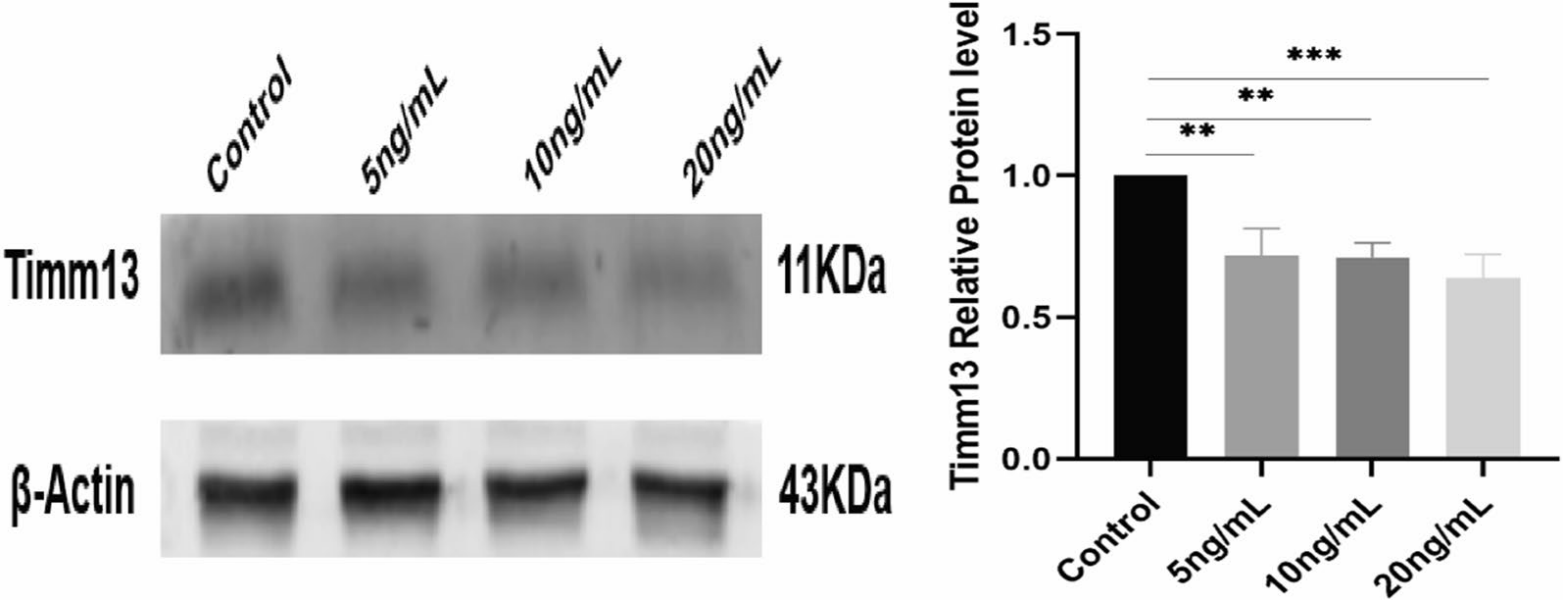
Validation of post-transcriptional levels in cell models. Expression levels of Timm13 in AML12 hepatocytes stimulated with different concentrations of TGF-β1

We detected Timm13 in hepatocytes at the protein level, and found significant downregulation of Timm13 protein in cell models (Fig. 7, P < 0.05). There was no significant correlation between the expression level of Timm13 and the concentration of TGF-β1 in the cell experiment (Fig. 7, P > 0.05).

### Silencing Timm13 reduced the expression of profibrogenic and apoptosis related genes

**Fig. 8:**
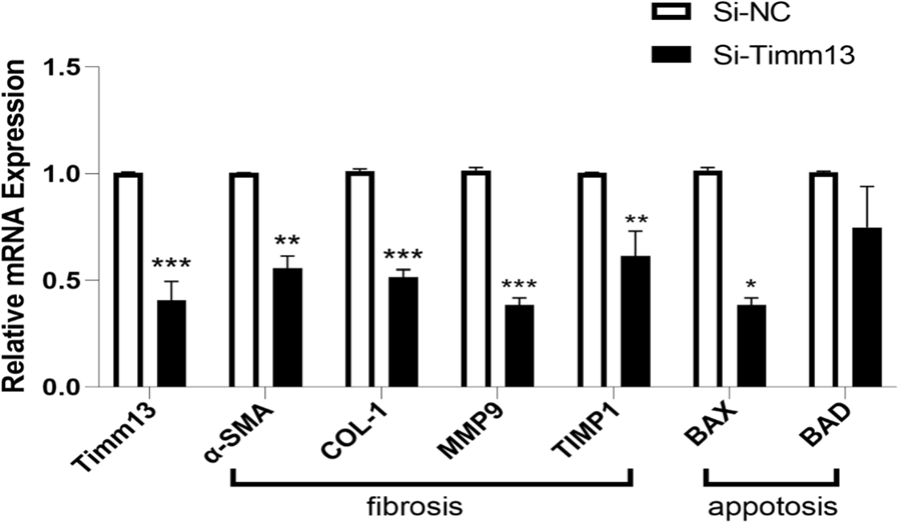
Silencing Timm13 reduced the expression of fibrosis and apoptosis genes. The expression of fibrosis genes (α-SMA, COL-1, MMP9, TIMP1) and apoptosis genes (BAX, BAD) decreased after silencing Timm13. (α-SMA, COL-1, MMP9, TIMP1, BAX, P < 0.05; BAD, P = 0.0813)

Hepatocytes (HCs) apoptosis is an important promoter of liver injury induced liver fibrosis. During the occurrence of liver fibrosis, the expression of profibrogenic genes and apoptosis related genes increased. In contrast, the expression of these genes decreased after silencing Timm13 (Fig. 8).

## Discussion

Liver fibrosis is considered to be a necessary stage in the progression of cirrhosis to hepatocellular carcinoma, in which fibrosis is a reversible stage [16]. However, there is still no effective clinical treatment to regulate the progress of liver fibrosis. Based on the above factors, we conducted the present study to identify significant DEGs between liver fibrosis and normal samples, and further verified that these differential genes were related to liver fibrosis. According to our previous research, mice began to show signs of liver fibrosis 2 weeks after CCl4 administration [14], and liver fibrosis could be reversed [16, 17]. Therefore, it is essential to find genes related to early liver fibrosis to provide targets for research and early intervention in liver fibrosis. The raw data were downloaded from GSE167033, and among a total of 21,722 genes, 178 DEGs were identified. The enrichment function results showed that these DEGs were mainly involved in mitochondria, nucleobase metabolic process, vascular endothelial growth factor receptor signaling pathway, cardiac conduction, regulation of glucose transmembrane transport, DNA replication, cytoplasmic microtubule organization, regulation of microtubule polymerization, and regulation of microtubule depolymerization. We then selected the most relevant genes via the PPI network and Cytoscape software. Finally, Timm13 was selected for further analysis. The liver is rich in mitochondria [5], and over the past decades, increasing studies have shown that mitochondrial function is related to liver fibrosis [18–21]. The translocation of nuclear-encoded mitochondrial preproteins is mediated by translocases in the outer and inner membranes [22, 23]. In the past ten years, research on mitochondrial inner membrane translocase has mainly focused on *Trypanosoma brucei* and yeast [24–27]. In addition, translocase of the mitochondrial inner membrane is also related to fungi and phospholipid metabolism [28, 29]. Timm13, a translocase of the mitochondrial inner membrane, is known to import and insert certain proteins into the mitochondrial inner membrane [30]. Timm13 cooperates with Timm8a in the space between mitochondrial membranes to promote the introduction of intimal substrate Timm23 [31]. A previous study showed that dental amalgam can reduce the expression of Timm13 in rat kidney [32]. Timm13 is associated with neuroblastoma [33], lung disease [34], nasopharyngeal carcinoma [35], hepatocellular carcinoma [36] and deafness/dystonia syndrome [37, 38]. Roesch et al. found that Timm8 and Timm13 are chaperones and are assembled in a 70 kDa complex, the deafness/dystonia protein 1/translocase of mitochondrial inner membrane 8a (DDP1/Timm8a) and Timm13 are also chaperones. When the DDP1/Timm8a-Timm13 complex assembly is defective, it can cause human deafness/dystonia syndrome [39]. Subsequently, a case report also analyzed the relationship between deafness/dystonia syndrome and DDP1/Timm8a-Timm13 [40]. In addition, it has been reported that the neurodegenerative disease Mohr–Tranebjaerg syndrome is caused by mutation of Timm8a, which forms a complex with Timm13, indicating that Timm13 is related to neurodegenerative diseases [41]. In a study of breast cancer, significant changes were found in the level of Timm13, but the specific mechanism was not clarified [42]. Interestingly, subsequent study showed that Timm13 highly predicted the overall survival (OS) and relapse-free survival (RFS) of basal breast cancer, and was identified as one of the essential genes for triple-negative breast cancer (TNBC) through transcriptomics. In this mechanism, the targeted knockdown of Timm13 reduced the proliferation potential of the cell models [43]. Shi et al. measured the changes in liver proteome and found that in the chicken, pork, beef and fish protein diet groups, the levels of translocase of inner mitochondrial membrane (Timm13, Timm8b and Timm9) were relatively low; thus, the meat protein diet could reduce the energy production level of the liver [44]. Recently, Kim et al. found that Timm13 showed reduced expression in human Alzheimer’s disease brain tissues [45]. The latest research demonstrated that the expression of Timm13 in cutaneous melanoma (SKCM) tissues was higher than that in adjacent tissues, and Timm13 expression was closely related to programmed cell death protein 1 (PD1), suggesting that it might regulate the tumor immune microenvironment and affect prognosis [46]. Research on the relationship between translocase of the mitochondrial inner membrane and various diseases has gradually increased and several articles have reported on Timm13 in the past two years. However, the relationship between translocase of the mitochondrial inner membrane or Timm13 and liver fibrosis has not been reported. Based on this study, Timm13 may have a profound influence on liver fibrosis. Mechanically, Timm13 regulates liver fibrosis by regulating the expression of hepatocytes profibrogenic and apoptosis related genes which is similar to the research results of Zhang et al. [47]; however, the specific mechanisms of Timm13 in liver fibrosis, such as regulatory pathways and specific sites, are still unclear. Thus, further investigation of the underlying mechanism of translocase of the mitochondrial inner membrane in liver fibrosis is urgently needed, which may facilitate the identification of a novel diagnosis and treatment or supplementary therapy regimens for liver fibrosis patients.

## Conclusions

The results of the present study showed that Timm13, a translocase of the mitochondrial inner membrane has a significant influence on liver fibrosis; however, the underlying mechanism has yet to be fully elucidated.

## Supplementary Information

**Additional file 1: Figure S1** Flowchart of the identification and verification of Timm13. The GSE167033 dataset was selected to identify differential genes, and common genes and their interacting proteins were analyzed by PPI, GO and KEGG analysis. The relationship between Timm13 and liver fibrosis was confirmed by an animal model and cell experiments. The mechanism of Timm13 on liver fibrosis was verified by a gene interference experiment. PPI, protein protein interaction; GO, gene ontology; KEGG, Kyoto gene and genome encyclopedia.

**Additional file 2: Table S1.** Information of GSE167033 dataset.

## Data Availability

The data used to support the findings of this study are included within the article.

## Abbreviations

Timm13: Translocase of the inner mitochondrial membrane 13
DEGs: Differentially expressed genes
PPI: Protein–protein interaction
STRING: Search Tool for the Retrieval of Interacting Genes/Proteins
HCs: Hepatocytes
HSCs: Hepatic stellate cells
CCl4: Carbon tetrachloride
GEO: Gene expression omnibus
RT-PCR: Real-time polymerase chain reaction
α- SMA: α-Smooth muscle actin
COL-1: Collagen I
BAX: BCL2-Associated X
BAD: Bcl-xL/Bcl-2asociated death promoter
MMP9: Matrix metalloprotein9
TIMP1: Tissue inhibitors of metalloproteinase 1
TGF-β1: Transforming growth factor beta1
ECM: Extracellular matrix
KEGG: Kyoto encyclopaedia of genes and genomes
GO: Gene ontology
DDP1: Deafness/dystonia protein 1
Timm8a: Translocase of mitochondrial inner membrane 8a
OS: Overall survival
RFS: Relapse free survival
TNBC: Triple-negative breast cancer
SKCM: Skin cutaneous melanoma
PD1: Programmed cell death protein 1
