# Supplementary figures and images for: Identification of Timm13 protein translocase of the mitochondrial inner membrane as a potential mediator of liver fibrosis based on bioinformatics and experimental verification
Source: J Transl Med. 2023 Mar 10;21:188. doi: 10.1186/s12967-023-04037-2 (PMC9999505; doi:10.1186/s12967-023-04037-2)

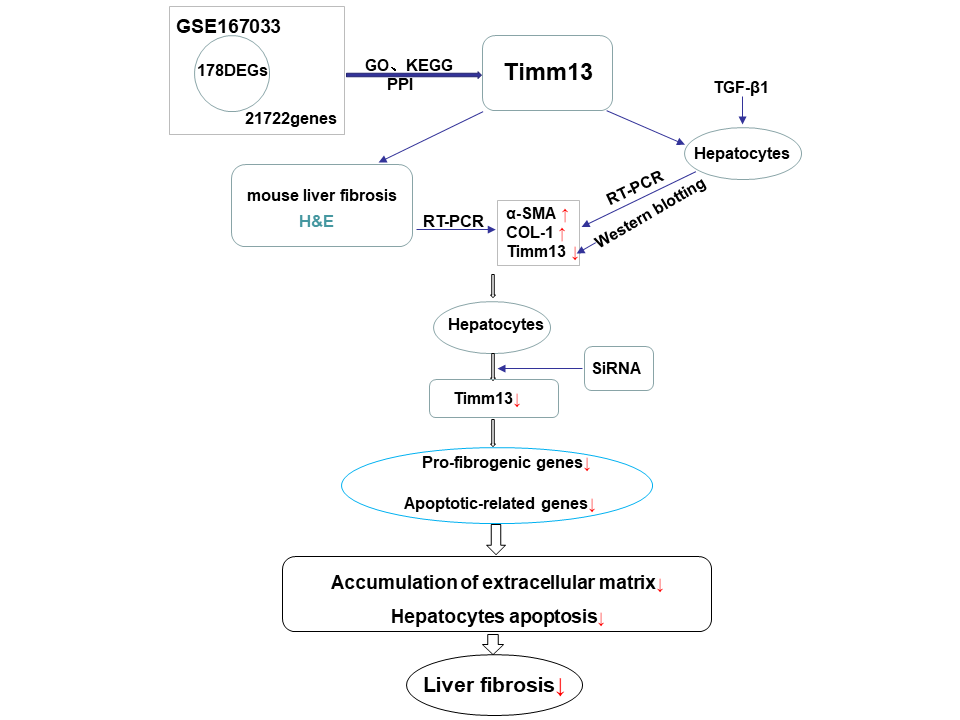

Supplement: Supplementary file 1 — Additional file 1: Figure S1 Flowchart of the identification and verification of Timm13. The GSE167033 dataset was selected to identify differential genes, and common genes and their interacting proteins were analyzed by PPI, GO and KEGG analysis. The relationship between Timm13 and liver fibrosis was confirmed by an animal model and cell experiments. The mechanism of Timm13 on liver fibrosis was verified by a gene interference experiment. PPI, protein protein interaction; GO, gene ontology; KEGG, Kyoto gene and genome encyclopedia. [file 12967_2023_4037_MOESM1_ESM.tif]
