# Supplementary material for: Identification of Timm13 protein translocase of the mitochondrial inner membrane as a potential mediator of liver fibrosis based on bioinformatics and experimental verification
Source: J Transl Med. 2023 Mar 10;21:188. doi: 10.1186/s12967-023-04037-2 (PMC9999505; doi:10.1186/s12967-023-04037-2)
Supplement: Supplementary file 2 — Additional file 2: Table S1. Information of GSE167033 dataset. [file 12967_2023_4037_MOESM2_ESM.doc]

**Table S1.** The specific information of GSE167033.

| Species | Sample | Num. samples | Treatment | Platform |
| --- | --- | --- | --- | --- |
| C57BL/6N Mice | Liver tissue | 46 | Intraperitoneal  Injection of CCl4 | GPL1261 |
